# Facial discoid lupus erythematosus complicated by a *Candida tropicalis* fungal granuloma in a patient with rhupus syndrome: a case report of successful treatment with oral itraconazole

**DOI:** 10.3389/fmed.2026.1897743

**Published:** 2026-09-03

**Authors:** Lingling Dong, Ting Zhang, Tong Hua, Yan Wang, Zhenhua Nie, Qingkang Lyu, Zuotao Zhao

**Affiliations:** 1 Department of Dermatology, Tianjin Academy of Traditional Chinese Medicine Afﬁliated Hospital, Tianjin, China; 2 Department of Dermatology, Tianjin Institute of Integrative Dermatology, Tianjin Academy of Traditional Chinese Medicine Afﬁliated Hospital, Tianjin, China; 3 Institute of Basic Research, Tianjin Academy of Traditional Chinese Medicine, Tianjin, China; 4 Immunology Center of Georgia, Augusta University, Augusta, GA, United States

**Keywords:** *Candida tropicalis*, case report, discoid lupus erythematosus, fungal granuloma, itraconazole

## Abstract

Discoid lupus erythematosus (DLE) is a chronic cutaneous subtype of lupus erythematosus characterized by inflammatory lesions that are highly susceptible to secondary infection, particularly in immunocompromised individuals. Cutaneous fungal granulomas caused by *Candida tropicalis* and superimposed on pre-existing DLE lesions are exceedingly rare and pose substantial diagnostic challenges. We report the case of a 48-year-old female patient with a 2-year history of rheumatoid arthritis (RA), who presented with facial lupus lesions in January 2024. During her disease course, she met the classification criteria for systemic lupus erythematosus (SLE), leading to a diagnosis of rhupus syndrome (RA-SLE overlap). After eight months of treatment with hydroxychloroquine and prednisone, her cutaneous lesions showed no improvement and instead progressed to nodules and pustules. Histopathological examination of a skin biopsy revealed classic features of DLE accompanied by granulomatous inflammatory infiltrates. Periodic acid–Schiff (PAS) staining and subsequent fungal culture confirmed *C. tropicalis* as the causative pathogen. The patient was ultimately diagnosed with DLE complicated by secondary *C. tropicalis* fungal granuloma in the setting of Rhupus syndrome. She received a 3-month course of combination therapy consisting of oral itraconazole (200 mg once daily) plus topical naftifine-ketoconazole cream, which resulted in complete clearance of all facial lesions. No recurrence was observed throughout a 12-month post-treatment follow-up period.

## Introduction

Rhupus syndrome is a rare overlap disorder characterized by the coexistence of clinical, serological, and imaging features of both rheumatoid arthritis (RA) and systemic lupus erythematosus (SLE) (1). The clinical manifestations of the two diseases typically appear sequentially rather than simultaneously, with RA-like features—particularly chronic, symmetric, and erosive polyarthritis—often predominating over SLE-related organ damage (2). Serologically, patients with rhupus syndrome frequently exhibit dual autoantibody profiles, including rheumatoid factor and anti-citrullinated protein antibodies characteristic of RA, alongside antinuclear antibodies, anti-double-stranded DNA, and anti-Smith antibodies typical of SLE. The SLE-related involvement in rhupus syndrome is generally mild and is most commonly manifested by hematological abnormalities and cutaneous, serosal, or renal involvement (3). DLE is a chronic form of cutaneous lupus erythematosus that typically presents as disk-shaped, erythematous, scaly plaques with sharp demarcation, dyspigmentation, atrophic scarring, and follicular hyperkeratosis, most commonly affecting sun-exposed areas such as the face, scalp, and ears. However, fungal infections superimposed on DLE lesions in patients with rhupus syndrome have rarely been documented.

In immunocompromised individuals, molds, yeasts, and dimorphic fungi may cause superficial, deep cutaneous, or disseminated invasive fungal diseases (4). Persistent infiltrative plaques and cutaneous ulcers should alert clinicians to the possibility of fungal granuloma. While the link between fungal infection and autoimmune rheumatic diseases has been well acknowledged, published reports detailing concurrent cutaneous fungal granuloma arising on pre-existing CLE or SLE lesions remain limited. Granulomas form as a protective host immune response against persistent pathogens including fungi and mycobacteria. These lesions consist of activated macrophages, epithelioid cells and multinucleated giant cells that sequester invading microorganisms; however, sustained antigen stimulation within granulomas also sustains chronic inflammation and progressive tissue damage. In immunocompromised patients with cutaneous lupus, opportunistic yeasts such as *C. tropicalis* may take advantage of impaired skin barrier function, invade the dermis and subcutaneous tissue, and induce chronic granulomatous inflammation (5).

Herein, we report a case of facial discoid lupus erythematosus complicated by a *Candida tropicalis* fungal granuloma in a patient with rhupus syndrome, which was successfully treated with oral itraconazole. This case highlights the importance of recognizing opportunistic fungal infections in cutaneous lupus lesions among patients with overlapping autoimmune syndromes.

## Case presentation

A 48-year-old woman presented in January 2024 with asymptomatic erythema and papules involving the forehead and bilateral malar regions, without an identifiable trigger. She denied a history of oral ulcers, alopecia, or photosensitivity. She reported no family history of autoimmune disease. She had a two-year history of rheumatoid arthritis (RA), which was well controlled with weekly methotrexate 10 mg. She was diagnosed with facial cutaneous LE at a local hospital and started on daily hydroxychloroquine (HCQ) 400 mg and prednisone 15 mg. After eight months of treatment, papules and nodules progressively increased in number within the erythematous lesions. In September 2024, new scattered bright-red papules emerged on her trunk and upper extremities, prompting further clinical evaluation, though her treatment regimen remained unchanged at that time.

**Figure 1:**
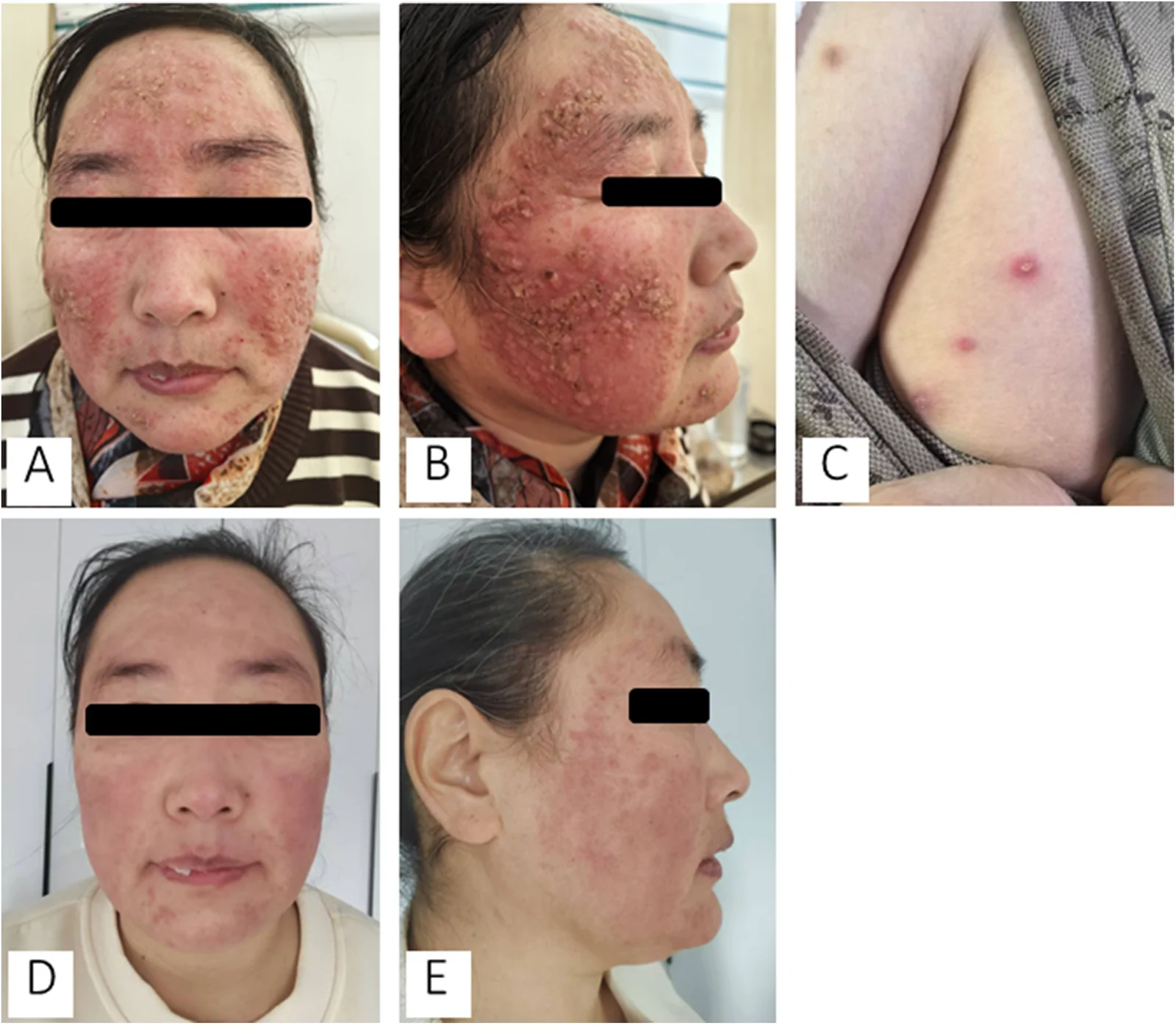
Symmetrical erythematous patches and plaques were observed on the face and forehead, characterized by overlying greasy, adherent scales. Arising from this erythematous background were numerous closely-set papules and nodules, which were firm to palpation and accompanied by localized areas of ulceration **(A,B)**. Scattered erythematous papules were also noted on the trunk and upper limbs **(C)**. Post-treatment, the majority of the papules and nodules on the face and forehead had subsided, with marked attenuation of the erythema **(D,E)**.

In October 2024, the patient presented to the dermatology department of our hospital. Dermatological examination revealed densely packed papules, nodules and pustules overlaying patchy erythema on the forehead and face. Several nodules coalesced, with central umbilication accompanied by superficial ulceration and crust formation. Scattered erythematous papules were distributed over the trunk and upper limbs; some lesions had partially resolved, leaving post-inflammatory hyperpigmentation and faint scarring (Figures 1A–C). Initial differential diagnoses included LE, infectious granuloma, pemphigus vegetans, and Kaposi's varicelliform eruption. The patient was admitted for comprehensive diagnostic workup and standardized management.

Upon admission, laboratory testing showed a normal white blood cell count of 5.28 × 10⁹/L and an elevated erythrocyte sedimentation rate (ESR) of 36 mm/h. Serological assays yielded positive antinuclear antibody (ANA) IgG at a titer of 1:320, alongside positive anti-SSA60 IgG, anti-SSB IgG and anti-cyclic citrullinated peptide (anti-CCP) IgG (Table 1). Antiphospholipid antibody screening was positive for anticardiolipin (aCL) IgG and anti-β2-glycoprotein I (aβ2GPI) IgG. Brain magnetic resonance imaging (MRI) displayed no remarkable abnormalities, whereas chest computed tomography (CT) identified fibrotic lesions in the right middle lobe and left upper lobe.

**Table 1:** Results of antinuclear antibody and antiphospholipid antibody panel.

Abbreviation	Test Item (IgG)	Result	Reference range	Unit
ANA	Antinuclear Antibody	1:320	<1:80	
ds-DNA	Double-stranded DNA Antibody	Negative (-)	<24	IU/mL
RNP	Ribonucleoprotein Antibody	Negative (-)	<10	IU/mL
Sm	Smith Antibody	Negative (-)	<10	IU/mL
SSA60	Sjögren's Syndrome-Related Antigen A (60 kDa) Antibody	Positive (+)	<10	IU/mL
SSB	Sjögren's Syndrome-Related Antigen B Antibody	Positive (+)	<10	IU/mL
Scl-70	Scleroderma-70 Antibody	Negative (-)	<10	IU/mL
Jo-1	Jo-1 Antibody (Histidyl-tRNA Synthetase)	Negative (-)	<10	IU/mL
rRNP	Ribosomal Ribonucleoprotein Antibody	Negative (-)	<10	IU/mL
PCNA	Proliferating Cell Nuclear Antigen Antibody	Negative (-)	<10	IU/mL
AHA	Anti-Histone Antibody	Negative (-)	<10	IU/mL
Ro52	Ro52 Antibody (TRIM21 Protein)	Negative (-)	<10	IU/mL
PM-Scl	Polymyositis-Scleroderma Antibody	Negative (-)	<10	IU/mL
ANuA	Anti-Nucleosome Antibody	Negative (-)	<10	IU/mL
CENP B	Centromere Protein B Antibody	Negative (-)	<10	IU/mL
AMA-M2	Antimitochondrial Antibody M2 Subtype	Negative (-)	<10	IU/mL
CCP	Cyclic Citrullinated Peptide Antibody	Positive (+)	<10	IU/mL
aCL	Anticardiolipin Antibody	Positive (+)	<10	IU/mL
aβ2GPI	Anti-Beta-2-Glycoprotein I Antibody	Positive (+)	<10	IU/mL

The following examinations returned normal or negative results: liver and renal function, procalcitonin, full coagulation panel including D-dimer, cytokine profiles (IL-2, IL-4, IL-6, IL-10, IL-12, IL-17, TNF-α, IFN-γ), pemphigus autoantibody panel (Dsg1, Dsg3, BP180, BP230), T-SPOT.TB, and serology for hepatitis B, hepatitis C, syphilis and human immunodeficiency virus (HIV). Routine urine and stool tests were unremarkable. Additional auxiliary examinations showed a normal electrocardiogram, mild pulmonary valve regurgitation on echocardiography, mild-to-moderate hepatic steatosis on abdominal ultrasound, and unremarkable renal ultrasound findings.

According to the 2019 European League Against Rheumatism/American College of Rheumatology (EULAR/ACR) classification criteria for systemic lupus erythematosus (SLE), the patient accumulated a total score of 12, thereby fulfilling the classification criteria for SLE. The score was derived from acute cutaneous lupus (facial lupus lesions), a history of joint involvement, and positive anticardiolipin (aCL) and anti-β2-glycoprotein I (aβ2GPI) antibodies. In addition, given the presence of positive anti-cyclic citrullinated peptide (anti-CCP) antibodies and a history of rheumatoid arthritis (RA) exceeding 2 years, the overall presentation meets the diagnostic definition of rhupus syndrome (RA-SLE overlap).

**Figure 2:**
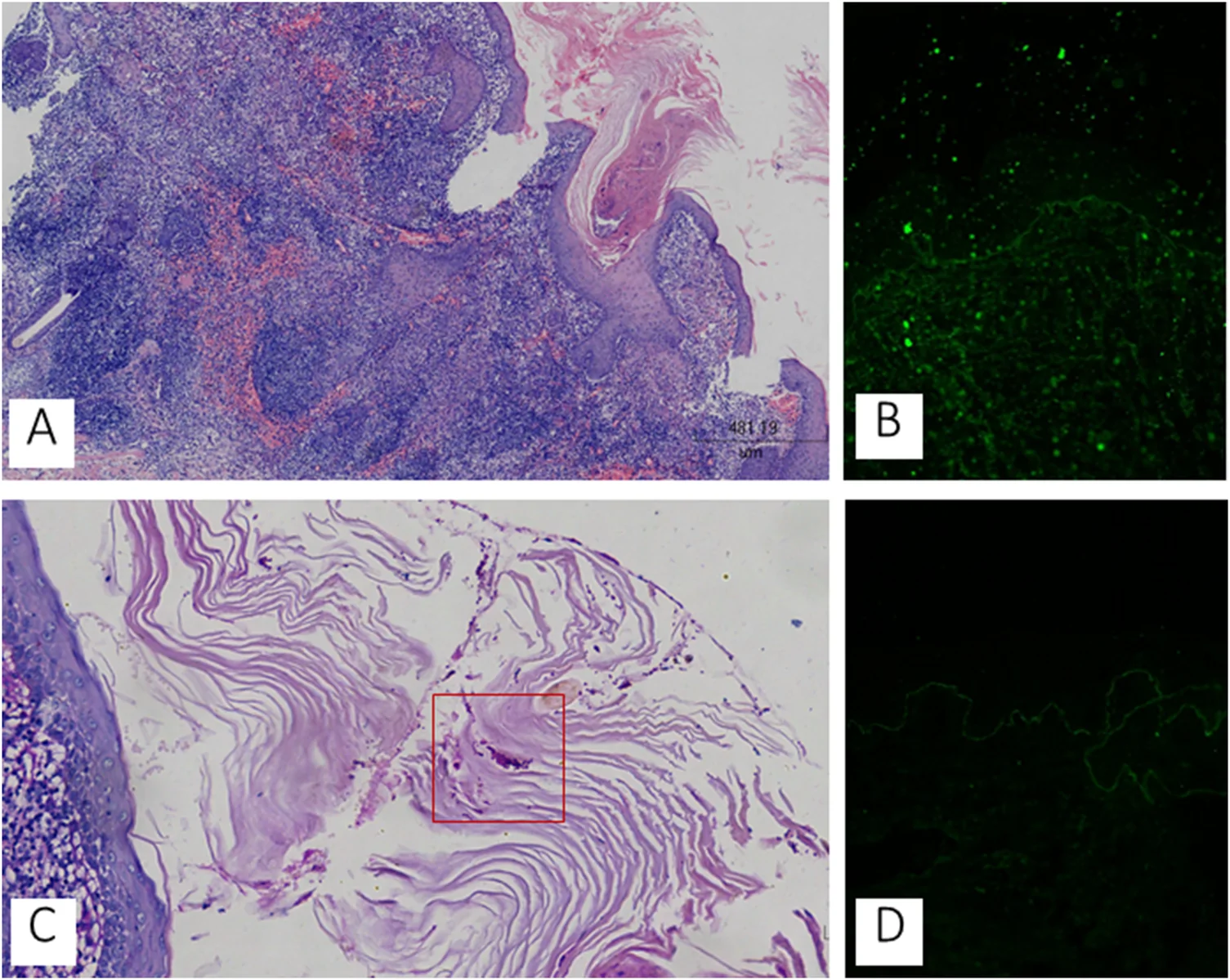
Histopathological examination of the facial biopsy specimen using hematoxylin-eosin staining demonstrated hyperkeratosis with associated parakeratosis, acanthosis with pseudoepitheliomatous hyperplasia, and prominent follicular plugging. The superficial dermis exhibited vascular proliferation, congestion, and focal hemorrhage. Within the dermis, a dense inflammatory infiltrate comprising lymphocytes, epithelioid cells, histiocytes, and sparse neutrophils was observed, accompanied by the formation of granulomas **(A)**. Direct immunofluorescence studies revealed linear deposition of IgG **(Panel B)** and complement C3 **(D)** along the dermoepidermal junction. Furthermore, Periodic acid–Schiff staining highlighted the presence of fungal spores localized within the stratum corneum **(C)**.

A skin biopsy was performed on a facial lesion. Hematoxylin and eosin (HE) staining demonstrated hyperkeratosis with parakeratosis, prominent follicular plugging, pseudoepitheliomatous hyperplasia. The dermis showed superficial vascular proliferation with congestion and focal hemorrhage, together with inflammatory cell infiltration extending throughout the dermis and involving the perifollicular and periadnexal regions. The inflammatory infiltration consisted of lymphocytes, neutrophils, epithelioid cells, and histiocytes, accompanied by granuloma formation (Figure 2A). Direct immunofluorescence (DIF) revealed weak linear deposition of IgG (±) and C3 (±) along the dermoepidermal basement membrane zone, while IgM and IgA staining were negative (Figures 2B,D). The findings of hyperkeratosis, prominent follicular plugging, inflammatory cell infiltration involving the full thickness of the dermis and surrounding the cutaneous appendages, along with positive DIF staining, strongly support the diagnosis of DLE. Meanwhile, superficial dermal vascular proliferation, congestion, and focal hemorrhage, in the absence of epidermal atrophy and dermal fibrosis, indicate that the DLE is in an active phase. The presence of parakeratosis, pseudoepitheliomatous hyperplasia, and dermal infiltration by lymphocytes, neutrophils, epithelioid cells, and histiocytes with granuloma formation can be observed in both hypertrophic DLE and infectious granuloma. Further Periodic acid–Schiff (PAS) staining visualized fungal spores within the stratum corneum (Figure 2C), supporting fungal involvement, and subsequent fungal culture confirmed the pathogen as *Candida tropicalis*. Accordingly, the definitive diagnosis was DLE complicated by a secondary fungal granuloma caused by C. tropicalis, in the setting of Rhupus syndrome.

Considering the opportunistic fungal infection attributable to the patient's long-term use of immunosuppressive agents, we tapered the prednisone dosage to 10 mg once daily (QD), temporarily discontinued methotrexate and hydroxychloroquine, and closely monitored the patient's relevant clinical symptoms and laboratory parameters. To address the fungal granulomatous lesions, oral itraconazole 200 mg once daily was initiated, together with topical naftifine-ketoconazole cream.

After two months of combination antifungal therapy, the facial erythema had markedly improved, and the papules, nodules, and pustules nearly completely resolved (Figures 1D,E). Consolidation antifungal treatment was continued for an additional month, after which both oral itraconazole and the topical antifungal cream were discontinued. After 2 months of antifungal therapy, following rheumatology clinic follow-up, hydroxychloroquine was re-initiated at 400 mg once daily (QD), and prednisone was tapered to 5 mg once daily (QD), with this regimen maintained to date. The patient's rhupus syndrome remains well controlled. At the 12-month post-treatment follow-up, the patient remained free of any recurrence of cutaneous lesions.

## Discussion

Rhupus syndrome is a rare overlap syndrome characterized by features of both rheumatoid arthritis and systemic lupus erythematosus. Patients frequently require prolonged immunosuppressive therapy, placing them at increased risk of opportunistic infections, including candidiasis. Glucocorticoids, often used in combination with conventional synthetic disease-modifying antirheumatic drugs, impair host antifungal immunity and increase the risk of candidiasis. Consistent with this, retrospective studies have shown that Candida species account for most secondary fungal infections in patients with SLE receiving long-term corticosteroid therapy (6). Among these pathogens, Candida tropicalis infection of pre-existing lupus lesions appears to be exceptionally rare. Other opportunistic fungi, including Mucorales, have also been reported in lupus-associated skin lesions (7, 8). These infections may progress rapidly and occasionally evolve into chronic fungal granulomas, making diagnosis and treatment challenging (9). To our knowledge, however, Candida tropicalis superinfection of a pre-existing DLE lesion has not previously been reported, highlighting the rarity of the present case.

Clinicians should suspect secondary infection when patients with cutaneous lupus erythematosus develop new, worsening, non-healing, or treatment-refractory skin lesions. In immunosuppressed patients, opportunistic fungal infections should be considered early in the differential diagnosis. Candida tropicalis typically infects skin with a disrupted barrier (10). However, it has also been reported to invade clinically intact skin in severely immunosuppressed individuals (10). In our patient, persistent facial erythema and epidermal disruption likely facilitated fungal invasion and the subsequent development of a secondary infection. Once a fungal infection is suspected, laboratory confirmation is essential. Skin biopsy, together with fungal stains (PAS and GMS), remains the cornerstone of diagnosis and should be complemented by pathogen culture and, when appropriate, molecular testing. Additional investigations, such as direct immunofluorescence and acid-fast staining, may help exclude alternative diagnoses. Together, these approaches enable an accurate diagnosis and guide appropriate treatment (9, 11).

Management of secondary cutaneous fungal granuloma in patients with rhupus syndrome is particularly challenging because effective infection control must be balanced against the need for ongoing immunosuppressive therapy, and evidence to guide treatment remains limited. Systemic antifungal therapy should be selected based on fungal species identification and *in vitro* antifungal susceptibility testing. Adjunctive topical antifungal therapy may further improve treatment outcomes (12). Previous reports have described complete lesion clearance and hair regrowth after eight weeks of combined oral griseofulvin and topical luliconazole therapy for cutaneous candidal granuloma (12). In parallel, immunosuppressive therapy should be adjusted according to disease activity while minimizing the risk of persistent infection. Hydroxychloroquine remains a cornerstone treatment for SLE and RA, but long-term therapy requires monitoring for ocular toxicity and QT interval prolongation (13). For refractory CLE, novel targeted therapies—including the approved anti-type I interferon receptor antibody anifrolumab, as well as investigational agents such as Janus kinase (JAK) inhibitors and the BDCA2-targeted monoclonal antibody litifilimab—have demonstrated prominent efficacy in improving cutaneous lesions (14, 15).

Patients with rhupus syndrome are particularly susceptible to opportunistic fungal infections because of chronic immunosuppression and impaired skin barrier function. Nevertheless, *Candida tropicalis* fungal granuloma complicating pre-existing DLE lesions has rarely been reported. This case highlights the importance of considering secondary fungal infection in treatment-refractory lupus skin lesions and demonstrates that early diagnosis together with appropriate antifungal therapy can result in a successful clinical outcome.

## Data Availability

The original contributions presented in the study are included in the article/Supplementary Material, further inquiries can be directed to the corresponding authors.

## References

[1] Li J Zhang J Li S-G Zhang L Zou Y Long T. Case report: triple autoimmune overlap: rheumatoid arthritis, systemic lupus erythematosus, and hypereosinophilic asthma with systemic manifestations. Front Immunol. (2025) 16:1659370. 10.3389/fimmu.2025.1659370 41624843 PMC12852425

[2] Amezcua-Guerra LM Roldán-Ortega J Mora-Ramírez M. Rhupus syndrome: current knowledge and future perspectives. Expert Rev Clin Immunol. (2025) 21(12):1709–24. 10.1080/1744666x.2025.2605662 41396200

[3] Antonini L Mauff LB Marcelli C Aouba A De Boysson H. Rhupus: a systematic literature review. Autoimmun Rev. (2020) 19(9):102612. 10.1016/j.autrev.2020.102612 32668290

[4] Ma Y Wang X Li R. Cutaneous and subcutaneous fungal infections: recent developments on host-fungus interactions. Curr Opin Microbiol. (2021) 62:93–102. 10.1016/j.mib.2021.05.005 34098513

[5] Yang X Jin X Yang Z Wang Y Wei A Yang X. Isolated cutaneous granuloma caused by Candida parapsilosis: case report and literature review. Mycopathologia. (2024) 189(2):20. 10.1007/s11046-023-00812-3 38407662

[6] Damara I Winston K Maulida F Ariane A. Factors associated with candidiasis in systemic lupus erythematosus patients in Cipto Mangunkusumo national general hospital: a single-center case-control study. Cureus. (2022) 14(7):e27107. 10.7759/cureus.27107 36000133 PMC9391667

[7] Li S Meng T Lin Y Xie D Li Z Jiang Q. Cutaneous mucormycosis in systemic lupus erythematosus: a case report. Front Immunol. (2025) 16:1602639. 10.3389/fimmu.2025.1602639 41000394 PMC12457325

[8] Wang Q Song Y Han D Cai H Yan Q Liu W. The first suspected disseminated Hormographiella aspergillata infection in China, diagnosed using metagenomic next-generation sequencing: a case report and literature review. Emerg Microbes Infect. (2023) 12(1):2220581. 10.1080/22221751.2023.2220581 37254739 PMC10259343

[9] Klein B Billi AC Abernathy-Close L Kahlenberg JM. Cutaneous lupus erythematosus—from pathogenesis to targeted therapy. Nat Rev Rheumatol. (2025) 21(12):703–18. 10.1038/s41584-025-01318-6 41204012 PMC12616405

[10] Gutiérrez Villanueva A Díaz de Santiago A Martínez Vera F De la Fuente Moral S. Candida tropicalis cellulitis in a patient with cirrhosis, well-controlled HIV and diabetes. BMJ Case Rep. (2021) 14(10):e241539. 10.1136/bcr-2020-241539 PMC8488749 34598954

[11] Vazquez T Patel J Kodali N Diaz DA Bashir MM Chin F. Plasmacytoid dendritic cells are not Major producers of type 1 IFN in cutaneous lupus: an in-depth immunoprofile of subacute and discoid lupus. J Invest Dermatol. (2024) 144(6):1262–72.e7. 10.1016/j.jid.2023.10.039 38086428 PMC12357323

[12] Peng Y Dong B Hu B Yang J Liu Z Tong Z. Adult Kerion caused by Trichophyton tonsurans. Mycopathologia. (2025) 190(1):17. 10.1007/s11046-024-00911-9 39841304

[13] Toumi E Bestion E Militello M Lepidi H Plauzolles A Bardin N. GNS561 (ezurpimtrostat), a small basic lipophilic molecule, prevents lupus phenotype in a pristane-induced lupus mouse model. Br J Pharmacol. (2025) 182(16):3786–99. 10.1111/bph.70058 40258389

[14] Chapman S Gold LS Lim HW. Janus kinase inhibitors in dermatology: part II. A comprehensive review. J Am Acad Dermatol. (2022) 86(2):414–22. 10.1016/j.jaad.2021.06.873 34228996

[15] Carter LM Wigston Z Laws P Vital EM. Rapid efficacy of anifrolumab across multiple subtypes of recalcitrant cutaneous lupus erythematosus parallels changes in discrete subsets of blood transcriptomic and cellular biomarkers. Br J Dermatol. (2023) 189(2):210–8. 10.1093/bjd/ljad089 36944572

